# Dr. Underwood's Treatise on the Diseases of Children; with Directions for the Management of Infants

**Published:** 1847-01

**Authors:** 


ART. I.—*Dr. Underwood's Treatise on the Diseases of Children; with Directions for the Management of Infants.* Tenth Edition, with Additions. By HENRY DAVIES, M.D., Senior Physician to the British Lying-in Hospital.—*London,* 1846. 8vo, pp. 596.

NONE of our readers, however old, or however young, but must be familiar with the title, at least, of this book. 'Underwood on Children' was the standing authority on its subject, in the days of our youth, and much pains have been taken, at different times, to keep the standard up to the level of the requirements of the passing day. Dr. Davies is the third editor and annotator of the book in our time,—Drs. Merriman and Marshall Hall having been his immediate predecessors. The present edition contains all the notes of these two gentlemen, as well as a large number of the editor's own, who moreover tells us that "in the conduct and production of the work he has received the assistance of the late Dr. Domeier, Dr. Klein Grant, and Dr. Sayer." Now, when it is considered that all, or nearly all the notes of the former editors, and all those of the present editor and his coadjutors, are *embodied in the text,* and that Dr. Davies, as he himself tells us, has, moreover, "deemed it advisable to adhere to Dr. Underwood's arrangement and, for the most part, also to retain his language and opinions," it will not surprise the reader, who will try to imagine a book so constructed, to learn, as he learns from the advertisement, that it contains "varieties of style and language, as well as some incongruities especially relative to the use of pronouns." And, sure enough, the pages of the volume do occasionally present such a composite character of structure, both as regards language and doctrines, as reminds us rather more of the coat of Joseph, than of the temples of Athens. We are sorry that Dr. Davies yielded to the wish of the publishers to have the original treatise reproduced, under any modification of plan and form, and did not devote the many precious hours that this mosaic must have cost him, to the task of giving us an original work on the same subject. This is a task for which his long experience had well prepared him, and which many of the excellent living notes he has here thrust into the carcase of old Underwood, prove he was well qualified for executing. But *dîs aliter visum*; the gods of Paternoster row and Princes street, doubtless, would not forego *the telling Title ;* and hence we are startled with yet another metempsychosis of the dear defunct partner of our youthful studies.

Nevertheless, and notwithstanding, we make the old friend with a new face welcome. With all its faults of form and substance, the book as it now stands is far from a bad book after all:—"unswept" as it is, and "besmeared with sluttish time," it contains a great deal of matter of real and substantial value, which will prove of permanent interest, not merely to the young, but to the old practitioner. With an amiable modesty which it would be well if some others could imitate, all that the editor says of his book is, that he "hopes that it will be found an improvement upon former editions, and a good practical treatise." We beg to assure him that he may justly take the credit of having done much more for the original, than the other editors did; and consequently, that, as it now stands, the book is, in our opinion, vastly superior to the preceding editions. Nay more—due allowance being made for its inherent and unavoidable defects,—we think Dr. Davies is entitled to claim for it the character of "a good practical treatise."

Dr. Davies has enriched the volume with many excellent practical observations of his own, and has drawn copiously from the stores of recent English writers. His own additions are all of a plain, unpretending practical character, conveying useful information much in the style in which Underwood himself would have given it. We regret that our present available space will not allow us to give even a single specimen of this new matter; but we can assure our readers that wherever they find the initials H. D. appended to an interpolated passage, they will find it worth perusal. By the way, it is a great defect in the volume that it is unprovided with an Index, as this is particularly needed in a book of such heterogeneous materials, and these so inartistically put together. A copious alphabetical index would have gone far to make up for the want of classification and arrangement inseparable from the plan on which the work has been composed. Among the many passages we had marked as bearing the stamp of Dr. Davies's own practical hand, we would refer the reader, for favorable specimens, to pages 186 and 313. He will there find excellent observations on two important diseases, "Spasm of the Glottis," and "Typhus or low fever of Children." Many others of a similar kind will strike the eye of the practised reader as he turns over the many-coloured pages now before our own; and we believe that few practical men whose business it is to treat the diseases of children, will fail, after even a slight examination, to add the volume to the shelf where he has deposited the works for daily reference in the exigencies of practice.

---

Art. II.—*Chemistry of the Four Seasons.* By Thomas Griffiths.—*London,* 1846. pp. 495.

